# Contrast-enhanced ultrasound features of focal pancreatic lesions in cats

**DOI:** 10.3389/fvets.2022.986948

**Published:** 2022-09-28

**Authors:** Silvia Burti, Alessandro Zotti, Giuseppe Rubini, Riccardo Orlandi, Paolo Bargellini, Federico Bonsembiante, Barbara Contiero, Mabel Marcuzzi, Tommaso Banzato

**Affiliations:** ^1^Department of Animal Medicine, Production and Health, University of Padua, Legnaro, Italy; ^2^ULTRAVET, Bologna, Italy; ^3^AniCura Tyrus Clinica Veterinaria, Terni, Italy

**Keywords:** focal pancreatic lesion, pancreatitis, pancreas, contrast-enhanced ultrasound, cytology, cat

## Abstract

A large overlap in the ultrasound (US) features of focal pancreatic lesions (FPLs) in cats is reported. Furthermore, only a small number of studies describing the contrast-enhanced ultrasound (CEUS) features of FPLs in cats have been conducted today. The aim of this study is to describe the B-mode US and CEUS features of FPLs in cats. Ninety-eight cats cytologically diagnosed with FPL were included. The lesions were classified as adenocarcinoma (*n* = 40), lymphoma (*n* = 11), nodular hyperplasia (*n* = 17), other benign lesion (OBL) (*n* = 20), cyst (*n* = 4) or other malignant lesion (OML) (*n* = 6). Several qualitative and quantitative B-mode and CEUS features were described in each case. OMLs and cysts were not included in the statistical analysis. A decision tree to classify the lesions based on their B-mode and CEUS features was developed. The overall accuracy of the cross-validation of the decision tree was 0.74 (95% CI: 0.63–0.83). The developed decision tree had a very high sensitivity and specificity for nodular hyperplasia (1 and 0.94, respectively) as well as good sensitivity and specificity for both adenocarcinomas (0.85 and 0.77, respectively) and OBLs also (0.70 and 0.93, respectively). The algorithm was unable to detect any specific feature for classifying lymphomas, and almost all the lymphomas were classified as adenocarcinomas. The combination between CEUS and B-mode US is very accurate in the classification of some FPLs, especially nodular hyperplasia and adenocarcinomas. Cytopathology and or histopathology is still a fundamental step FPL diagnostic workflow.

## Introduction

Signs of pancreatic disease in cats are usually non-specific, often making the diagnosis of pancreatic disorders challenging. Pancreatitis is by far the most common pathology affecting this organ (1), while neoplasia has an overall low incidence in cats (2). Degenerative changes in the pancreas (e.g., nodular hyperplasia) are also common, especially in older animals (3). The workflow for diagnosing pancreatic disease in cats includes: a complete blood work-up, specific biochemical tests (e.g., feline pancreatic lipase immunoreactivity concentration), and abdominal ultrasonography, followed by a computed tomography (CT) scan and/or histopathology or cytopathology (1, 3). Feline pancreatic lipase immunoreactivity concentration is often used as tool for diagnosis of pancreatic disease but, recent studies reveal that it can be elevated both in pancreatitis and pancreatic neoplastic disease in cats (3). Even if the accuracy of all the above tests is still debated (4) and, therefore, achieving a definitive diagnosis of pancreatic disease is still challenging.

Contrast-enhanced ultrasonography (CEUS) has gained increasing popularity as an ancillary diagnostic imaging technique in cats in recent years, but the restricted number of reports, along with the small number of subjects usually included in those reports, is still acting as a limiting factor in making this imaging technique widespread. The normal and the pathological CEUS features of several different organs have been reported in cats (5–8). To the best of the authors' knowledge, the only available comprehensive study on the pathological pancreas is by Rademacher et al. (9). The contrast-enhanced, power and color doppler appearance of 15 cases of pathological pancreas were described in the study: 8 of these were diagnosed as inflammation, 4 as nodular hyperplasia, and 3 as neoplasia (1 lymphoma, 1 neuroendocrine carcinoma and 1 adenocarcinoma of the exocrine pancreas). Vascularization and blood volume were significantly higher in all the cases, with pancreatitis-associated tumors confirmed in 85% of pathological pancreas. Nonetheless, the CEUS features of the individual histopathological lesions were not described in the study and, therefore, are not immediately usable by the clinician (9). Cervone et al. reported a case of insulinoma in a cat, explored both with B-mode ultrasound and CEUS examination: the three identified nodules all appeared as hypoechoic and hypoenhancing lesions (10).

It seems evident that the CEUS features of feline pancreatic diseases have remained an almost unexplored field in veterinary medicine to date and, therefore, the aim of this paper is to describe the B-mode US and CEUS features of the most common focal pancreatic lesions (FPLs) in cats. To this end, a large number of cases were collected from three different veterinary institutions and described in a standardized fashion. Furthermore, a decision tree, based on the CEUS and B-mode features, to help the clinician in the classification of the FPL has been developed.

## Methods

### Cases

Ninety-eight cats referred to AniCura Tyrus Veterinary Clinic (Tyrus Veterinary Clinic, Via A. Bartocci 1/G, Terni, Italy), Ultravet (Ultravet, Via E. Fermi 59, San Giovanni in Persiceto, Bologna, Italy) and the Veterinary Teaching Hospital at the University of Padua (O.V.U.D., Viale dell'Università 16, Padova, Italy) for specialty CEUS and cytopathological examination of the pancreas, between January 2008 and June 2021, were retrospectively selected by GR and SB. Only cats with both CEUS and cytopathological examinations were included. Cats with abdominal effusion, ongoing chemotherapy, or having no final cytopathological diagnosis were excluded. Complete signalment and medical history were recorded for each cat.

All the methods were applied in accordance with the relevant guidelines and regulations. This study was conducted respecting Italian Law N° 26/2014 (that transposes the EU directive 2010/63/EU). As the data used in this study were collected during routine clinical activity, no ethical committee approval was needed. Informed consent for personal data processing was obtained from the owners.

### B-mode ultrasound and CEUS

A complete B-mode ultrasonographic examination of the abdomen and a CEUS examination of the pancreas were performed on each animal by two veterinarians (GR and PB, with 19 and 16 years' experience in small animal ultrasonography, respectively). Three different ultrasound scanners (GE Logic E9 [GE Medical Systems], Esaote MyLab70 Gold [Esaote Italia] or Esaote Twice [Esaote Italia]) were used.

An 8-h fasting period prior to examination was respected for each cat, and sedation with butorphanol tartrate (10) was administered intramuscularly in restless cases. Sonovue^®^ (Bracco Imaging BV, Geneva, Switzerland) was administered intravenously at the dose of 0.05 ml/kg, followed by a 5-ml saline flush through a three-way stopcock with an extension tube (containing 0.5 ml of fluid) directly connected to the intravenous cannula (5). Each lesion was scanned continuously for at least 1 min, or until the end of the wash-out phase. The mechanical index was set to a low value (0.02). B-mode features were evaluated using both videos and still images, depending on the availability, whereas all CEUS scans were evaluated in video mode.

### Qualitative and quantitative analysis

All the B-mode and CEUS examinations were simultaneously reviewed by the same two veterinarians (GR and SB). The interpretation was concorded following a consensus discussion. The following qualitative B-mode features of the lesions were evaluated: (1) focal or diffused lesion; (2) the presence of a pseudocystic appearance, described as such if the lesion had a well-defined capsule and evident intralesional echoes or structures (present or absent); (3) echogenicity (anechoic, hypoechoic, isoechoic or hyperechoic); (4) acoustic enhancement (present or absent); (5) peripancreatic reactivity (present or absent). The lesion was classified as anechoic if no echoes were detectable. If echoes were present, the echogenicity was compared to that of the ultrasonographically normal pancreatic parenchyma or to the nearby tissues, and defined as hypoechoic, isoechoic or hyperechoic. The echogenicity of the lesion was defined as mixed when both hypoechoic and hyperechoic areas were detected. Peripancreatic reactivity was defined as hyperechogenicity of the peripancreatic fat eventually associated with the presence of peripancreatic fluid. The maximum diameter of the focal lesions was also evaluated during B-mode examinations. When the pancreatic tissue was diffusely involved, the maximum pancreatic thickness was recorded.

The following qualitative CEUS features were evaluated during the wash-in phase: (1) enhancement degree (hyperenhancing, isoenhancing, hypoenhancing or non-enhancing) compared to the normal pancreatic parenchyma or to the peripancreatic fat (if sonographically normal); (2) distribution of the contrast medium (diffused, peripheral or central); (3) intralesional microcirculation (present or absent); (4) homogeneity (homogeneous or inhomogeneous); (5) hypoperfused areas (present or absent). Intralesional microcirculation was defined as the presence of hyperenhancing vessels inside the lesion. The following qualitative features were evaluated during the wash-out phase: (1) enhancement degree compared to the normal pancreatic parenchyma or to the peripancreatic fat (if sonographically normal), with classification as having no wash-out for (a) lesions that were hyperenhancing during the wash-in phase and isoenhancing during wash-out and (b) lesions that were isoenhancing or hypoenhancing during both wash-in and wash-out, while (c) lesions that were hyperenhancing or isoenhancing during the wash-in phase and hypoenhancing during wash-out were classified as having hypoenhancing wash-out; (2) homogeneity (homogeneous or inhomogeneous); (3) pattern of contrast-medium decrease (centripetal, centrifugal or diffuse).

The following quantitative CEUS features of the lesions were evaluated: (1) time to enhancement (TTE), which was the timespan between injection and appearance of the signal in the lesion; (2) time to peak (TTP), as the timespan between injection and the peak intensity of the signal; (3) time to wash-in (TTWI), calculated as TTP minus TTE.

The qualitative features of the individual lesions were classified after consensus discussion. More specifically, after simultaneous evaluation of B-mode and CEUS examinations, a consensus discussion between the two evaluators was used to assign the final features.

To reduce digital storage consumption, all the CEUS examinations were stored as AVI files and the original digital imaging and communication in medicine (DICOM) files were no longer available. The time-intensity curves used to extract the quantitative CEUS features from the AVI files were calculated using a custom-built MATLAB script developed by one of the authors (TB).

### Cytopathological examinations

Pancreatic lesions were sampled by means of US-guided fine-needle aspiration. A May-Grünwald-Giemsa stain was used. A trained clinical pathologist, specifically appointed for this task, performed classification of the lesions based on previously published criteria (11, 12).

### Statistics and data analysis

Statistical analysis was performed using a commercially available software (SAS 9.4, by SAS Institute Inc., Cary, NC, USA). Descriptive statistics were generated and the Shapiro-Wilk test was used to assess the normality hypothesis for all continuous data. Continuous data were reported as mean (±standard deviation) if normally distributed, and median (interquartile range) for non-normally distributed data. Comparisons among the 5 groups (adenocarcinoma, insulinoma, nodular hyperplasia, cyst, and abscess) were performed using a chi-squared test (Fisher's exact test when appropriate) for categorical variables, the Kruskal–Wallis for non-parametric variables, and one-way ANOVA for normally distributed ones. *Post-hoc* pairwise comparisons were performed using Dwass-Steel-Critchlow-Fligner adjustment or Bonferroni correction for non-normally or normally distributed data, respectively.

The decision tree was created to assist the clinician in the interpretation of such a complex examination. A recursive partitioning method was used with the rpart package of R—https://cran.r-project.org/web/packages/rpart/vignettes/longintro.pdf. A three-step procedure was adopted to build the decision tree: (1) the features providing the best data splitting were selected; (2) the tree was pruned to the lowest number of branches and the lowest misclassification rate (13) using a 10-fold cross-validation method; (3) a confusion matrix was built by reapplying the decision tree to classify all the cases in the database, and the values of actual vs. predicted samples (obtained from the decision tree classification) were compared. Sensitivity, specificity, positive and negative predictive value, and balanced accuracy were calculated.

## Results

### Cytopathological examination

The 98 cases were divided into six groups, based on the results of the cytopathological analysis: 40 adenocarcinomas, 20 other benign lesions (OBLs: 9 inflammatory cysts, 4 abscesses, 3 serous cystadenomas, 1 adenoma, 1 cystadenoma, 1 granuloma, 1 lipoma), 17 nodular hyperplasia, 11 lymphomas, 6 other malignant lesions (OMLs: 2 mesenchymal neoplasia, 1 neuroendocrine neoplasia, 1 insulinoma, 1 metastasis from melanoma, 1 histiocytic sarcoma), and 4 cysts.

### B-mode ultrasound and CEUS

The B-mode and CEUS features are described here below and are listed in Tables 1–4. Due to the limited number of cases, the OML and cyst categories were not included in the statistical analysis. Significant differences in the distribution among pathological groups were evident for all the B-mode features, except for peripancreatic reactivity. Most (18/20) of the OBLs had a pseudocystic appearance (**Figures 4**, **5**) even if only 5 of the 23 lesions displaying such a feature were adenocarcinomas. Interestingly, almost all (19/20) of the OBLs displayed a mixed echogenicity even if the same echogenicity was evident in all the other pathological categories. Likewise, acoustic enhancement was evident in most (19/20) OBLs, but this feature was shared with all the pathological categories.

**Table 1:** Qualitative and quantitative B-mode features of the lesions, along with cytopathological classification.

	**Adenocarcinoma (*n* = 40)**	**Lymphoma (*n* = 11)**	**Nodular hyperplasia (*n* = 17)**	**Other benign lesion^▴^(*n* = 20)**		Cyst (*n* = 4)*	Other malignant lesion^Δ^ (*n* = 6)*
**B-mode features**					***p*-value**		
Pseudocystic appearance^+^ (*n* = 23)	5 (12%) ^b^	0 ^b^	0 ^b^	18 (90%)^a^	< 0.001	0	0
Echogenicity*					< 0.001		
hypoechoic^+^ (*n* = 40)	16 (40%)^b^	7 (64%)^ab^	15 (88%)^a^	1 (5%)^c^	< 0.001	0	1 (17%)
mixed^+^ (*n* = 52)	23 (57%)^b^	4 (36%)^bc^	2 (12%)^c^	19 (95%)^a^	< 0.001	0	4 (66%)
hyperechoic (*n* = 2)	1 (3%)	0	0	0	–	0	1 (17%)
Isoechoic	–	–	–	–		4 (100%)	0
Anechoic	–	–	–	–		0	0
Presence of acoustic enhancement^+^ (*n* = 62)	23 (57%)^b^	9 (82%)^ab^	3 (18%)^c^	19 (95%)^a^	< 0.001	4 (100%)	4 (66%)
Presence of peripancreatic reactivity^+^ (*n* = 57)	20 (50%)	7 (64%)	8 (47%)	16 (80%)	0.112	3 (75%)	3 (50%)

Different letters along rows mean significant different value for p < 0.05.

^*^Fisher's exact test.

^+^k-proportion test.

^▴^Other benign lesion (OBL): 7 inflammatory cysts, 6 abscesses, 3 serous cystadenomas, 1 adenoma, 1 granuloma, 1 cystadenoma, 1 lipoma.

^Δ^Other malignant lesion (OML): 2 mesenchymal neoplasia, 1 neuroendocrine neoplasia, 1 insulinoma, 1 metastasis, 1 sarcoma.

^*^Data not included in the statistical analysis.

**Table 2:** Qualitative CEUS wash-in features of the lesions, along with cytopathological classification.

	**Adenocarcinoma (*n* = 40)**	**Lymphoma (*n* = 11)**	**Nodular hyperplasia (*n* = 17)**	**Other benign lesion^▴^(*n* = 20)**		Cyst (*n* = 4)*	Other malignant lesion^Δ^ (*n* = 6)*
**CEUS wash-in**					***p*-value**		
Enhancement degree*					< 0.001		
Hyperenhancement^+^ (*n* = 42)	20 (50%)^a^	7 (64%)^a^	0^b^	14 (60%)^a^	< 0.001	0	5 (83%)
Hypoenhancement^+^ (*n* = 24)	19 (47%)^a^	3 (27%)^ab^	0^b^	1 (5%)^b^	< 0.001	0	1 (17%)
Isoenhancement^+^ (*n* = 23)	1 (3%)^b^	1 (9%)^b^	17 (100%)^a^	5 (20%)^b^	< 0.001	0	0
Non-enhancing (*n* = 4)	0	0	0	0	–	4 (100%)	0
Distribution*					0.004		
Diffuse^+^ (*n* = 68)	27 (68%)^b^	9 (82%)^ab^	17 (100%)^a^	10 (50%)^b^	0.007	0	5 (83%)
Peripheral^+^ (*n* = 25)	13 (32%)	2 (18%)^ab^	0^b^	9 (45%)^a^	0.014	0	1 (17%)
Central (*n* = 1)	0	0	0	1 (5%)	–	0	0
Presence of intralesional microcirculation^+^ (*n* = 40)	27 (67%)^a^	6 (55%)^ab^	0^c^	3 (15%)^bc^	< 0.001	0	4 (67%)
Homogeneity^+^ (*n* = 58)	20 (50%)^b^	4 (36%)^b^	17 (100%)^a^	15 (70%)^ab^	0.001		2 (33%)
Hypoperfused areas^+^ (*n* = 51)	22 (55%)^a^	6 (54%)^a^	0^b^	18 (90%)^a^	< 0.001		5 (83%)

Different letters along rows mean significant different value for p < 0.05.

^*^Fisher's exact test.

^+^k-proportion test.

^▴^Other benign lesion (OBL): 7 inflammatory cysts, 6 abscesses, 3 serous cystadenomas, 1 adenoma, 1 granuloma, 1 cystadenoma, 1 lipoma.

^Δ^Other malignant lesion (OML): 2 mesenchymal neoplasia, 1 neuroendocrine neoplasia, 1 insulinoma, 1 metastasis, 1 sarcoma.

^*^Data not included in the statistical analysis.

**Table 3:** Quantitative CEUS wash-in features of the lesions, along with cytopathological classification.

	**Adenocarcinoma (*n* = 40)**	**Lymphoma (*n* = 11)**	**Nodular hyperplasia (*n* = 17)**	**Other benign lesion^▴^(*n* = 20)**		Cyst (*n* = 4)*	Other malignant lesion^Δ^ (*n* = 6)*
					***p-*value**		
Max dimension^§^ (mm)	1.8 (1.3–2.8)^a^	1.6 (1.1–2.7)^a^	0.8 (0.5–0.9)^b^	1.9 (1.3–3.6)^a^	< 0.001	0.5 (0.5–0.6)	1.7 (1.4–3.4)
TTE^§^ (sec)	5.0 (4.0–7.0)	4.0 (4.0–7.0)	6.0 (5.0–8.0)	5.0 (4.0–6.0)	0.404		5.5 (5.0–6.0)
TTP^§^ (sec)	9.0 (8.0–11.0)	8.0 (7.0–13.0)	12.0 (9.0–15.0)	14.0 (8.0–16.0)	0.028		10.0 (9.3–10.8)
TTWI^§^ (sec)	4.0 (3.0–5.0)	4.0 (2.0–6.0)	5.0 (4.0–9.0)	7.0 (4.0–9.0)	0.056		5.0 (3.5–5.8)

^§^Kruskal–Wallis test; data are presented as median (interquartile range).

^▴^Other benign lesion (OBL): 7 inflammatory cysts, 6 abscesses, 3 serous cystadenomas, 1 adenoma, 1 granuloma, 1 cystadenoma, 1 lipoma.

^Δ^Other malignant lesion (OML): 2 mesenchymal neoplasia, 1 neuroendocrine neoplasia, 1 insulinoma, 1 metastasis, 1 sarcoma.

^*^Data not included in the statistical analysis.

Note: Within each row, data that do not share a superscript letter are significantly different based on the post hoc comparison test.

**Table 4:** Qualitative CEUS wash-out features of the lesions, along with cytopathological classification.

	**Adenocarcinoma (*n* = 40)**	**Lymphoma (*n* = 11)**	**Nodular hyperplasia (*n* = 17)**	**Other benign lesion^▴^(*n* = 20)**		Cyst (*n* =4 )*	Other malignant lesion^Δ^ (*n* = 6)*
CEUS wash-out					***p-*** **value**		
Enhancement degree*					0.002		
No wash-out^+^ (*n* = 70)	27 (67%)^b^	5 (45%)^b^	17 (100%)^a^	17 (85%)^ab^	0.005		4 (67%)
Hypoenhancement^+^ (*n* = 24)	13 (32%)^a^	6 (54%)^a^	0^b^	3 (15%)^ab^	0.005		2 (33%)
Hyperenhancement (*n* = 0)					–		
Homogeneity^+^ (*n* = 14)	8 (61%)	2 (33%)	–	3 (100%)	0.153		1 (50%)

Different letters along rows mean significant different value for p < 0.05.

^*^Fisher's exact test.

^+^k-proportion test.

^▴^Other benign lesion (OBL): 7 inflammatory cysts, 6 abscesses, 3 serous cystadenomas, 1 adenoma, 1 granuloma, 1 cystadenoma, 1 lipoma.

^Δ^Other malignant lesion (OML): 2 mesenchymal neoplasia, 1 neuroendocrine neoplasia, 1 insulinoma, 1 metastasis, 1 sarcoma.

^*^Data not included in the statistical analysis.

Even if several of the B-mode features of the FPLs showed statistically significant differences, none was specifically associated to any of the included cytopathological categories. In fact, even if most (18/23) of the lesions showing a pseudocystic appearance were OBLs, 5 were adenocarcinomas. Interestingly, most (52) of the focal lesions showed a mixed echogenicity even if the lesions showing a mixed echogenicity were found distributed among different histopathological groups. Acoustic enhancement was evident in almost all the OBLs (not surprisingly, since 18/19 OBLs had a pseudocystic appearance). Peripancreatic reactivity was evident in a large number (57) of cases, regardless of cytopathological group.

Significant differences were also evident for most of the CEUS qualitative features, with the exception of distribution of contrast medium. Among the quantitative features, only the maximum dimension showed statistically significant differences. The results of the statistical analysis are reported in Tables 2–4.

Example images of the B-mode and CEUS features of all the cytopathological categories of lesions are depicted in Figures 1–8. Description of both the B-mode and CEUS features of each considered cytopathological category is discussed below.

**Figure 1:**
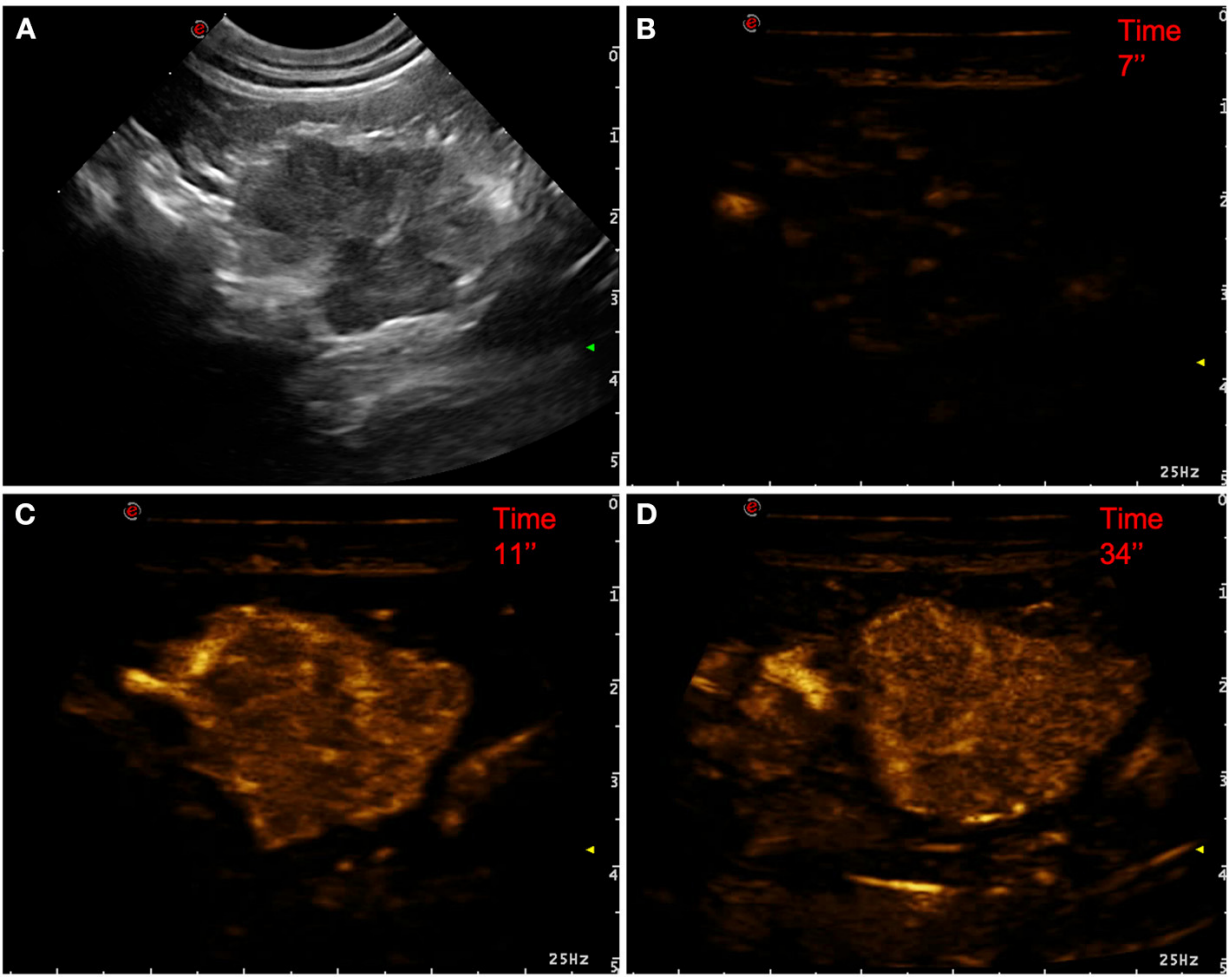
Example of an adenocarcinoma showing: no pseudocystic appearance, mixed echogenicity, absence of acoustic enhancement, presence of peripancreatic reactivity at US; hyperenhancement, diffused and inhomogeneous distribution of contrast medium, presence of intralesional microcirculation, absence of hypoperfused areas during wash-in at CEUS; no wash-out. **(A)** Image obtained from US examination; **(B)** image obtained at TTE; **(C)** image obtained at TTP; **(D)** image obtained during wash-out.

**Figure 2:**
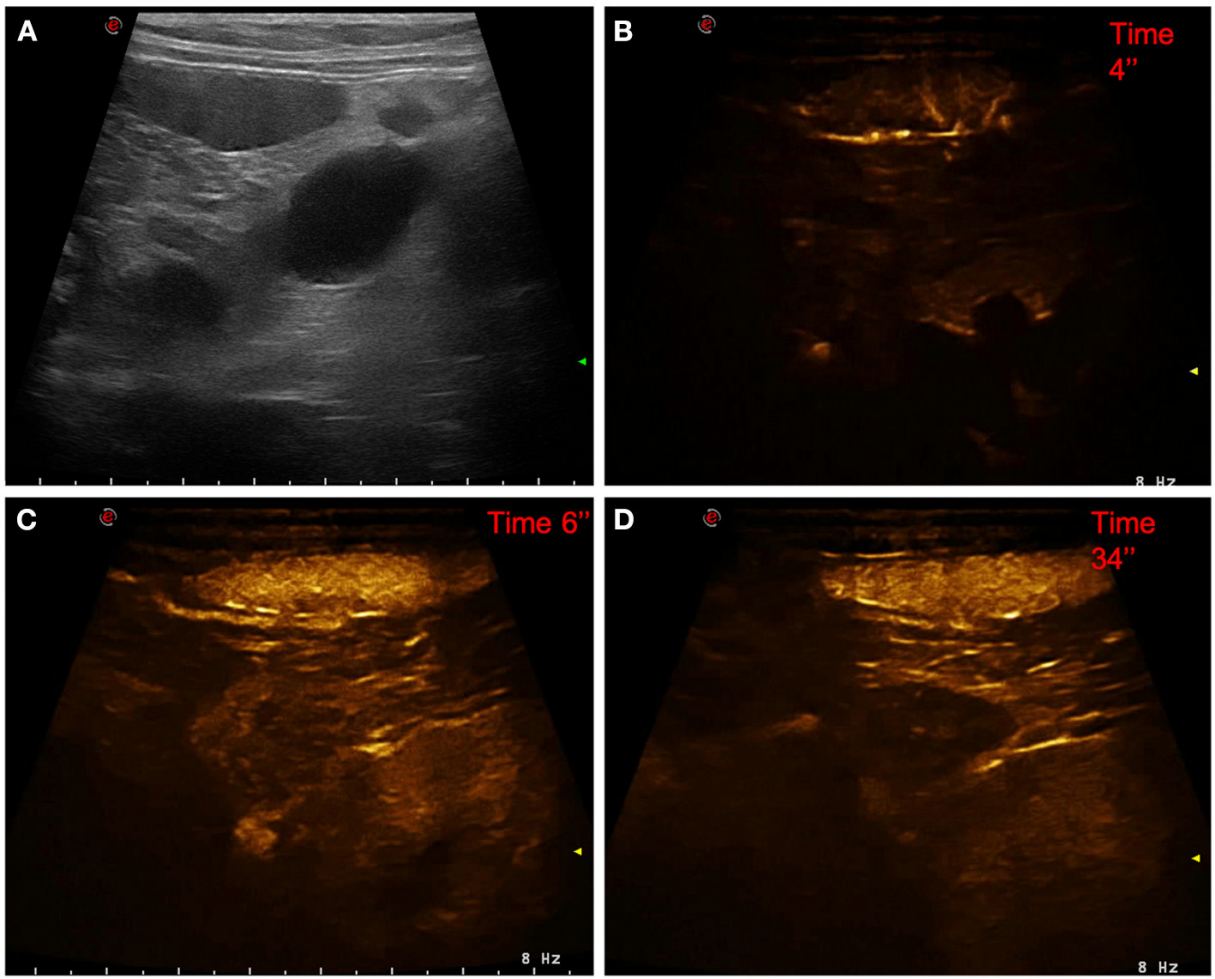
Example of a lymphoma showing: no pseudocystic appearance, hypoechogenicity, presence of acoustic enhancement, and presence of peripancreatic reactivity at US; hyperenhancement, diffused and inhomogeneous distribution of contrast medium, absence of intralesional microcirculation, and absence of hypoperfused areas during wash-in at CEUS; hypoenhancing, inhomogeneous and diffused wash-out. **(A)** Image obtained from US examination; **(B)** image obtained at TTE; **(C)** image obtained at TTP; **(D)** image obtained during wash-out.

**Figure 3:**
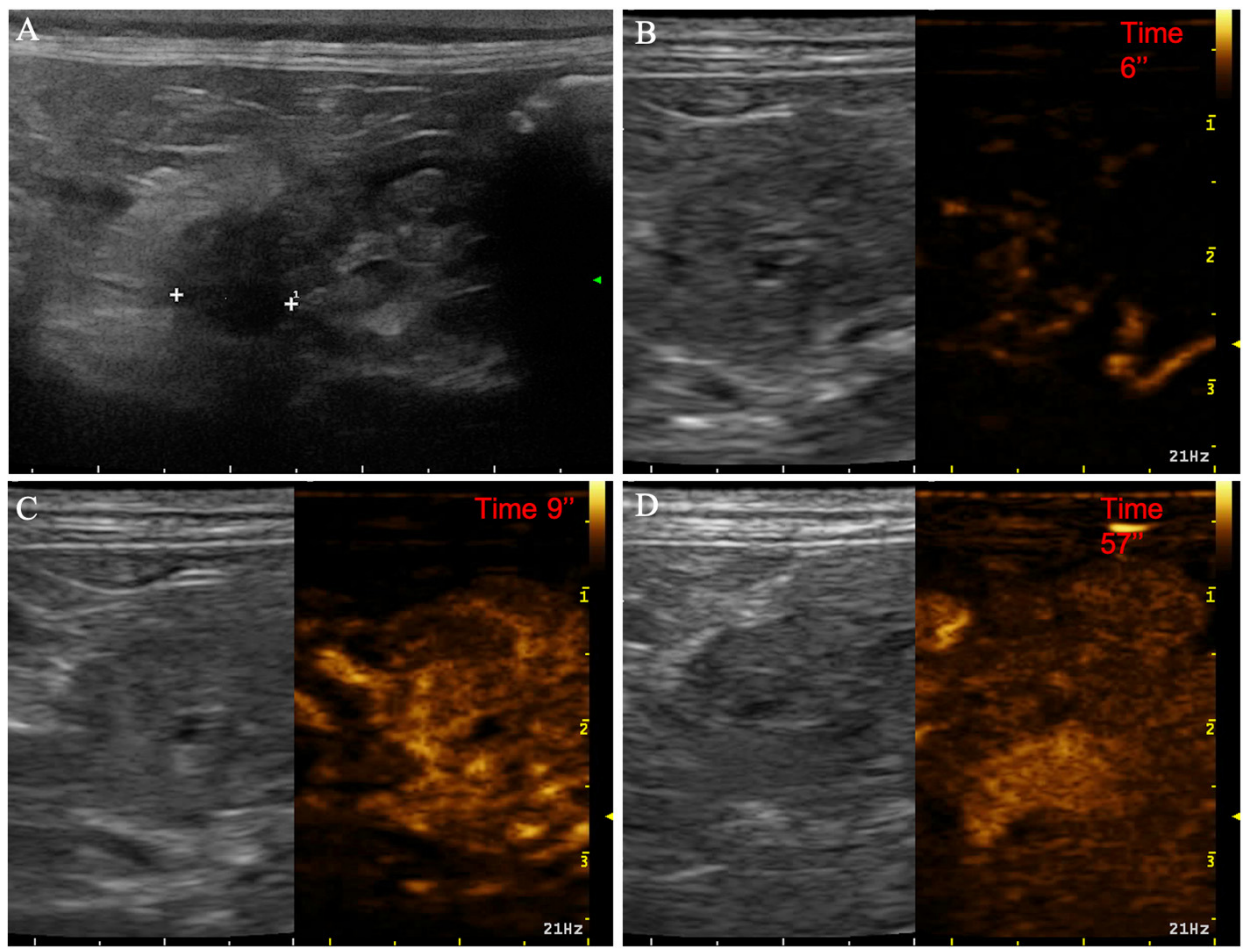
Example of nodular hyperplasia showing: no pseudocystic appearance, hypoechogenicity, absence of acoustic enhancement, and presence of peripancreatic reactivity at US; isoenhancement, diffused and homogeneous distribution of contrast medium, absence of intralesional microcirculation and hypoperfused areas during wash-in at CEUS; no wash-out. **(A)** Image obtained from US examination; **(B)** image obtained at TTE; **(C)** image obtained at TTP; **(D)** image obtained during wash-out.

**Figure 4:**
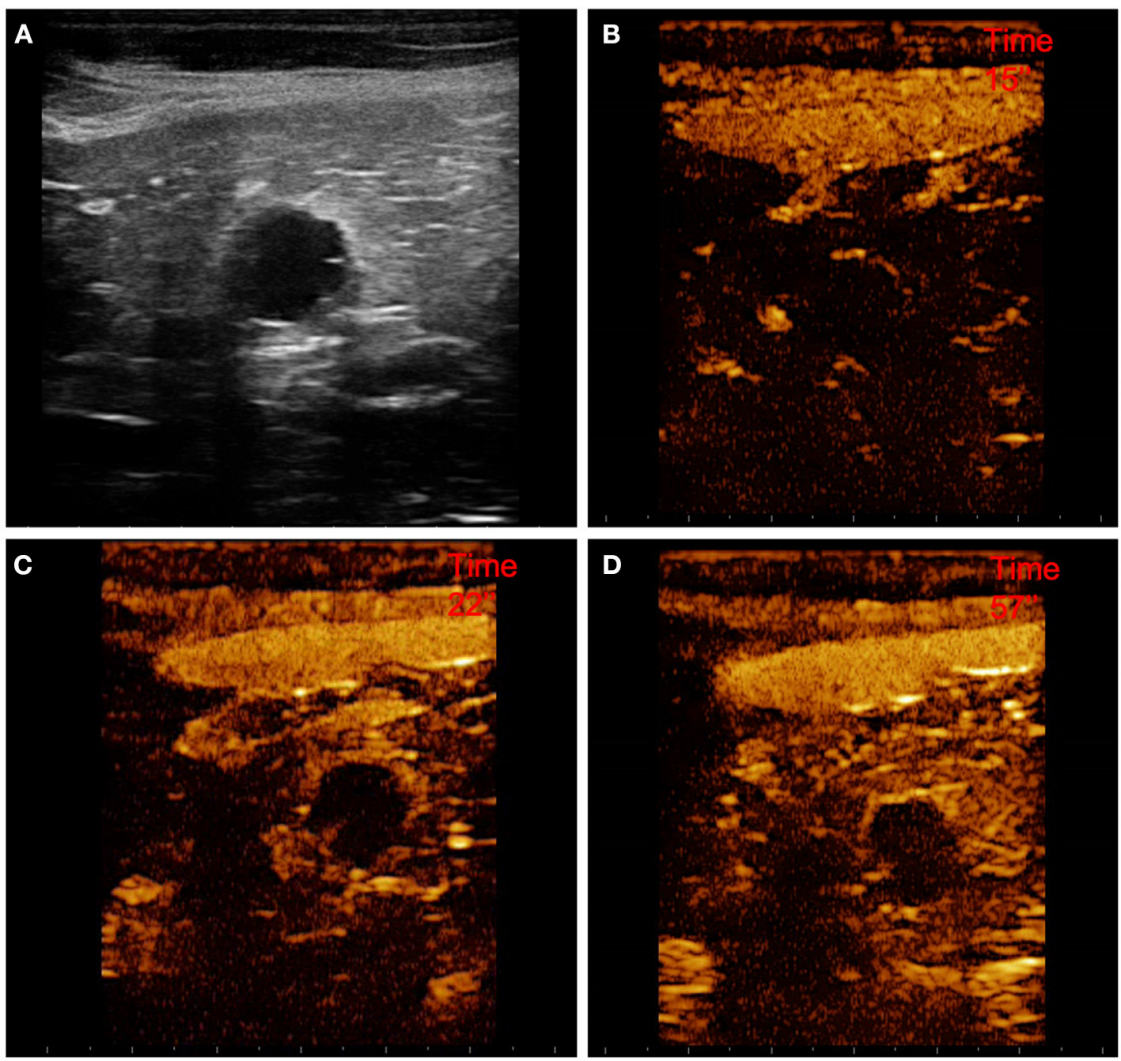
Example of a cystadenoma (OBL) showing: pseudocystic appearamce, mixed echogenicity, presence of acoustic enhancement, and presence of peripancreatic reactivity at US; hyperenhancement, peripheral and homogeneous distribution of contrast medium, presence of intralesional microcirculation, and presence of hypoperfused areas during wash-in at CEUS; no wash-out. **(A)** Image obtained from US examination; **(B)** image obtained at TTE; **(C)** image obtained at TTP; **(D)** image obtained during wash-out.

**Figure 5:**
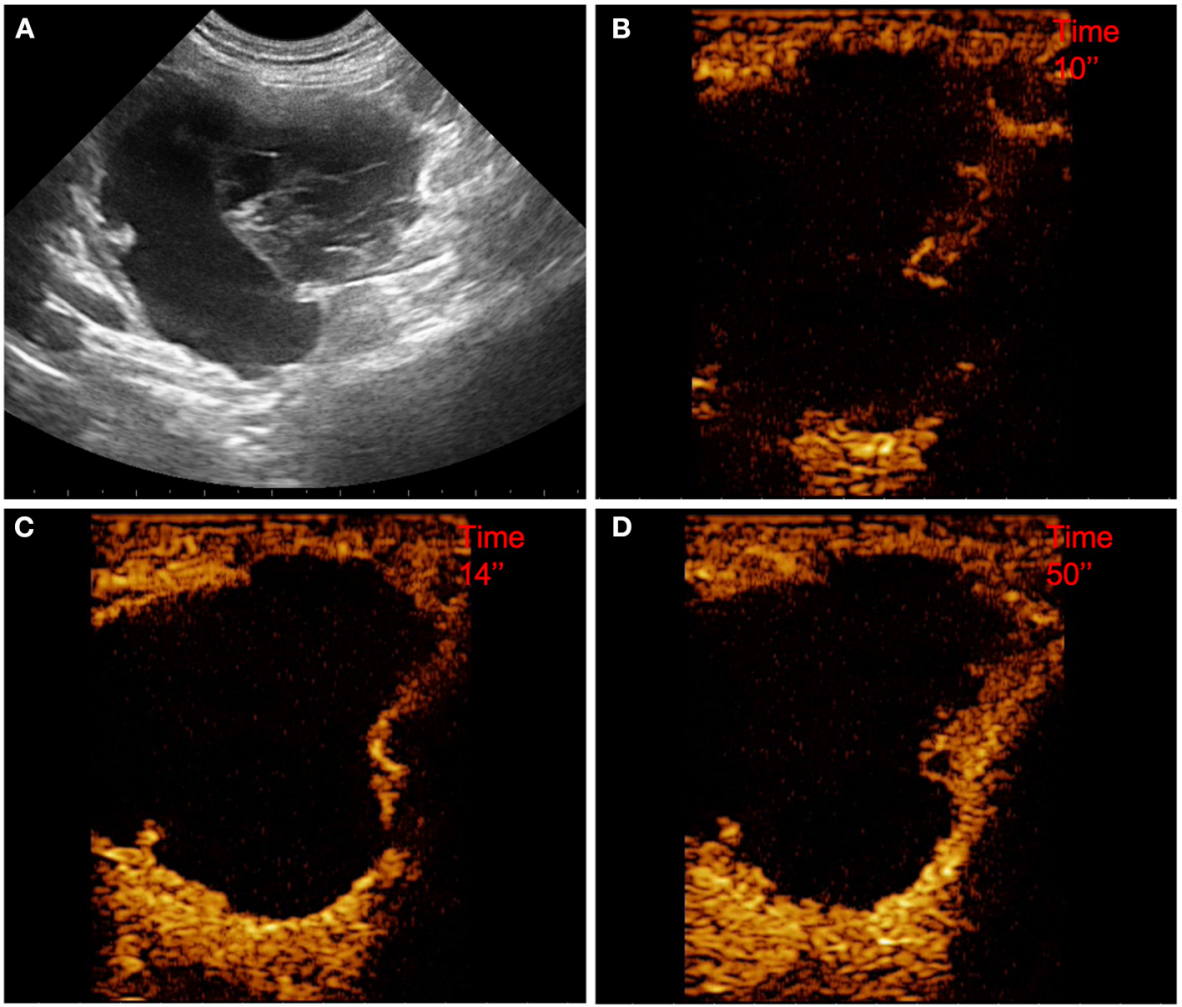
Example of a pseudocyst (OBL) showing: pseudocystic appearance, mixed echogenicity, presence of acoustic enhancement, and presence of peripancreatic reactivity at US; hyperenhancement, peripheral and homogeneous distribution of contrast medium, absence of intralesional microcirculation, and presence of hypoperfused areas during wash-in at CEUS; no wash-out. **(A)** Image obtained from US examination; **(B)** image obtained at TTE; **(C)** image obtained at TTP; **(D)** image obtained during wash-out.

**Figure 6:**
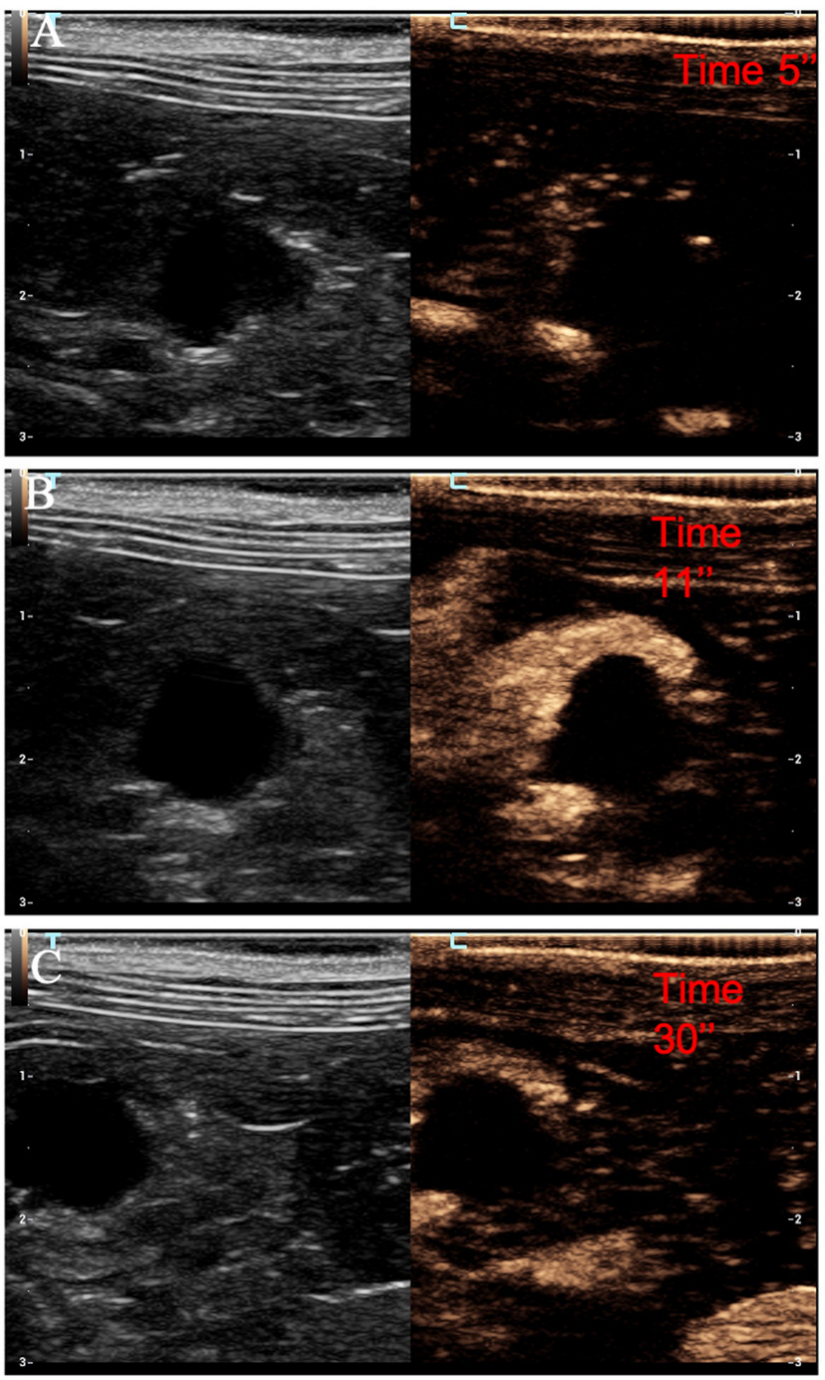
Example of a cyst showing: no pseudocystic appearance, anechoic lesion, presence of acoustic enhancement, and presence of peripancreatic reactivity at US; non-enhancing at CEUS. **(A)** Image of the surrounding pancreatic parenchyma obtained at TTE; **(B)** image of the surrounding pancreatic parenchyma obtained at TTP; **(C)** image of the surrounding pancreatic parenchyma obtained during wash-out.

**Figure 7:**
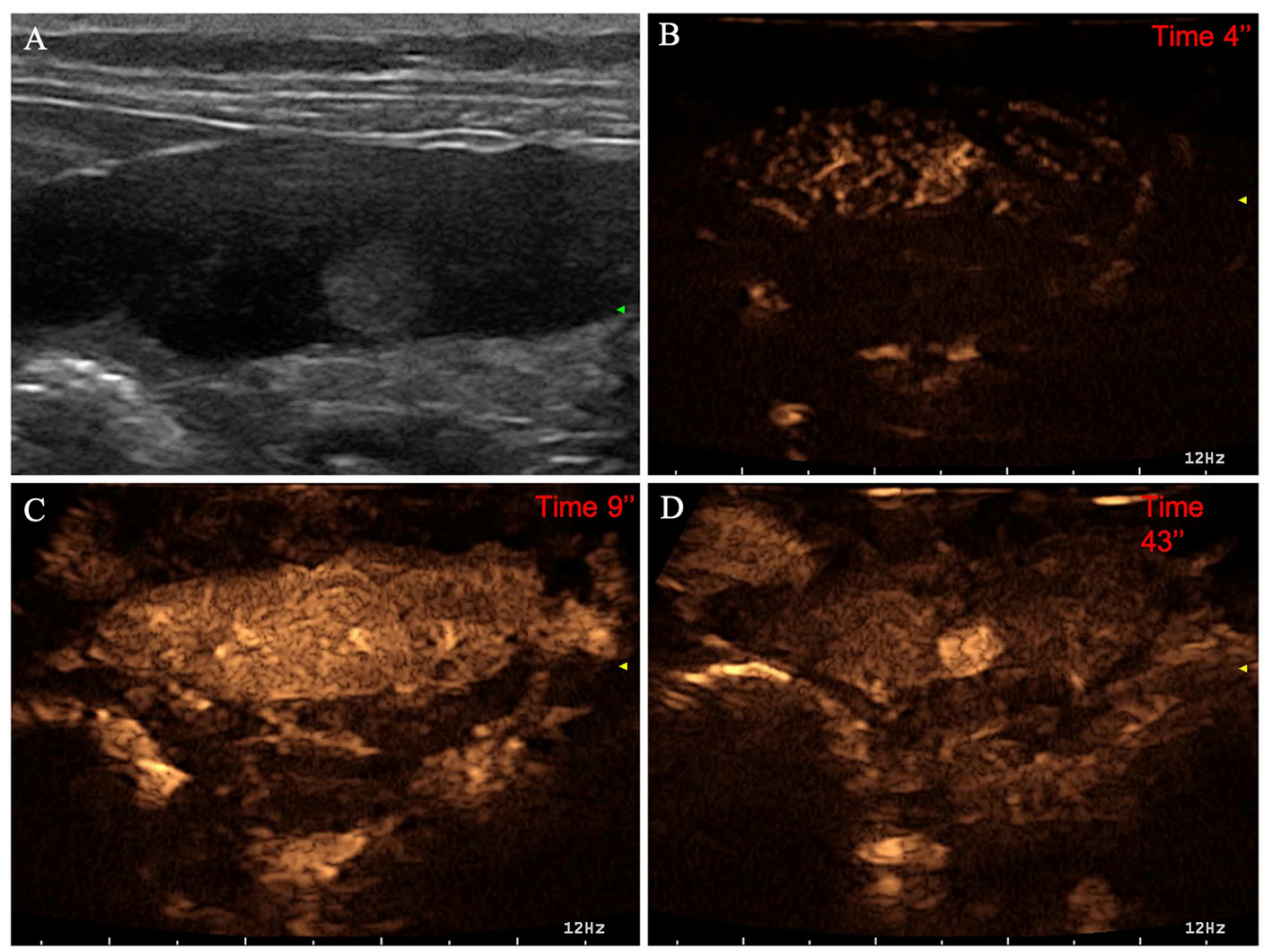
Example of an insulinoma (OML) showing: no pseudocystic appearance, hyperechogenicity, absence of acoustic enhancement, and presence of peripancreatic reactivity at US; hyperenhancement, diffused and homogeneous distribution of contrast medium, absence of intralesional microcirculation, and absence of hypoperfused areas during wash-in at CEUS; no wash-out. **(A)** Image obtained from US examination; **(B)** image obtained at TTE; **(C)** image obtained at TTP; **(D)** image obtained during wash-out.

**Figure 8:**
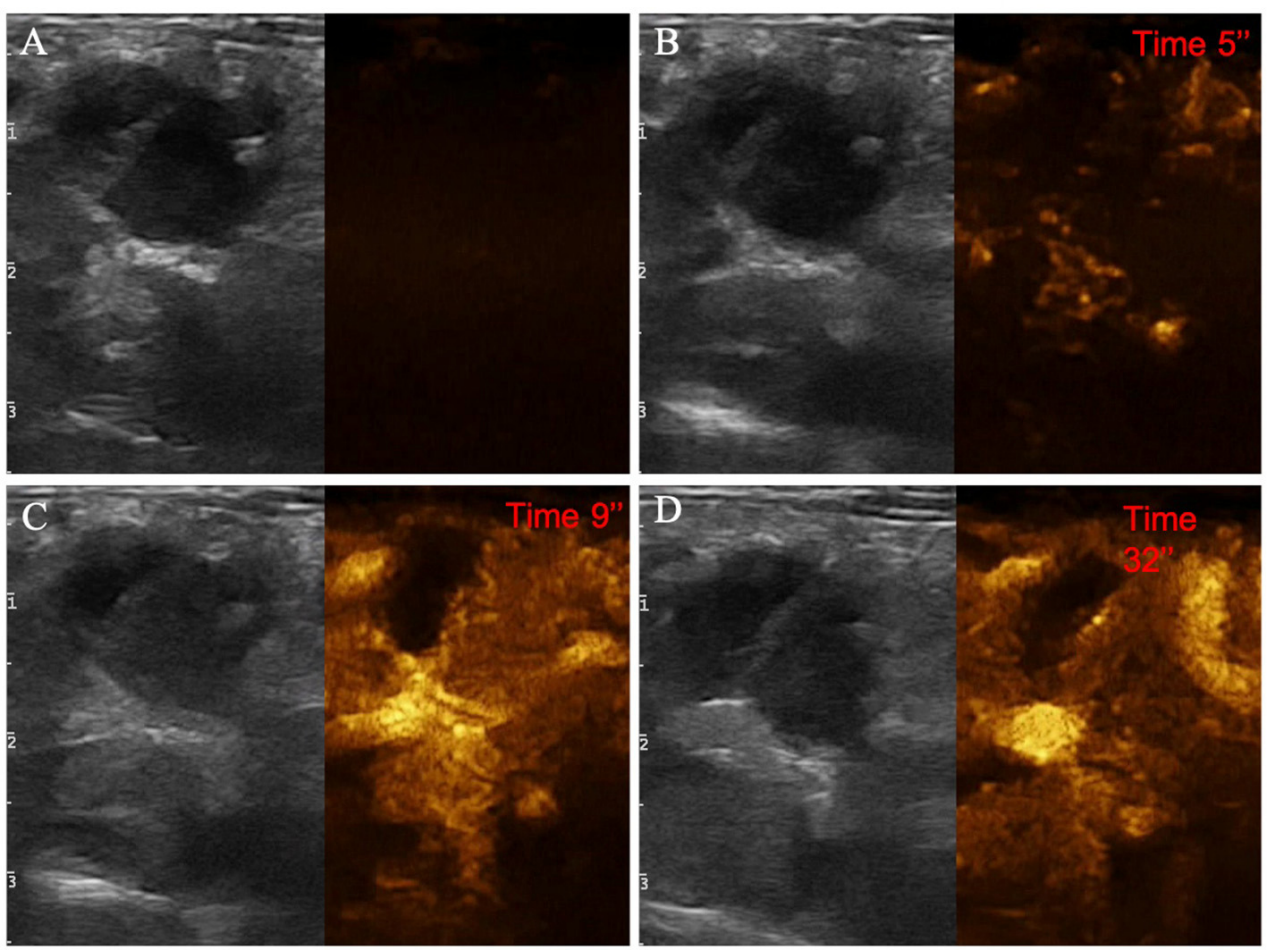
Example of an adenocarcinoma showing: pseudocystic appearance, mixed echogenicity, presence of acoustic enhancement, and absence of peripancreatic reactivity at US; hypoenhancement, diffused and inhomogeneous distribution of contrast medium, presence of intralesional microcirculation, and presence of hypoperfused areas during wash-in phase at CEUS; hypoenhancing, inhomogeneous and diffused wash-out. **(A)** Image obtained from US examination; **(B)** image obtained at TTE; **(C)** image obtained at TTP; **(D)** image obtained during wash-out.

**Figure 9:**
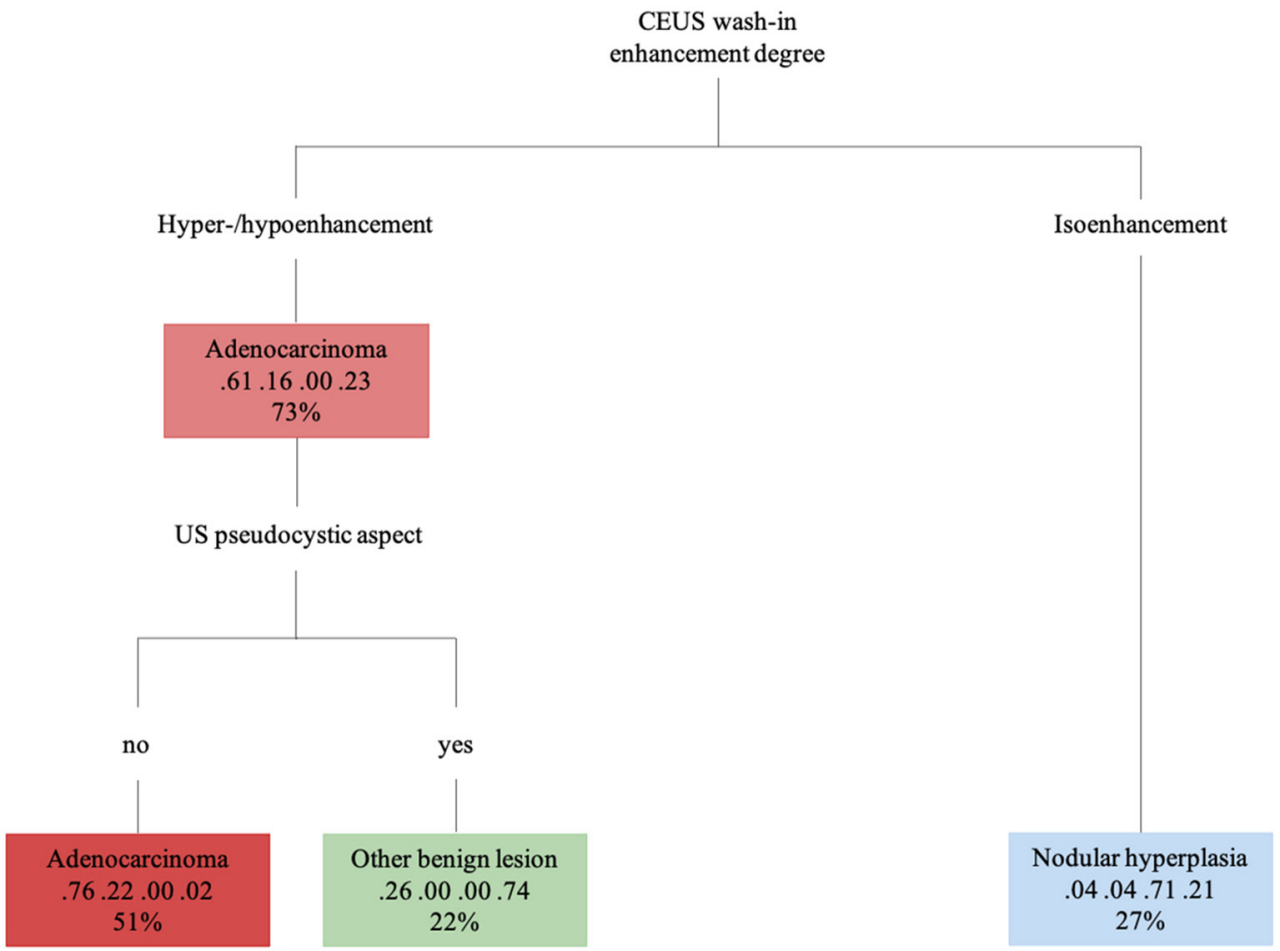
The machine learning-based decision tree developed on the qualitative and the quantitative CEUS features of the focal pancreatic lesions. The probability of each class at a specific node and the percentage of observations used at that node are reported as the second and third lines in each box, respectively.

The decision tree resulting from the analysis is depicted in Figure 9. The confusion matrix and the sensitivity, specificity, positive predictive value, negative predictive value and balanced accuracy for each of the included categories, as resulting from the cross-validation of the decision tree, are reported in Tables 5, 6, respectively. The overall accuracy of the cross-validation of the decision tree was 0.74 (95% CI: 0.63–0.83). The algorithm was unable to detect any specific feature for classifying lymphomas, but almost all the lymphomas were classified as adenocarcinomas.

**Table 5:** Confusion matrix that summarizes the performance of the machine learning-based decision tree, giving the number of predicted cases.

	**Actual**			
**Predicted**	**Adenocarcinoma**	**Lymphoma**	**Nodular hyperplasia**	**Other benign lesion**
Adenocarcinoma	34	10	0	1
Lymphoma	0	0	0	0
Nodular hyperplasia	1	1	17	5
Other benign lesion	5	0	0	14

**Table 6:** Results of the classification of the pancreatic lesions based on the machine learning-based decision tree.

	**Adenocarcinoma**	**Lymphoma**	**Nodular hyperplasia**	**Other benign lesion**
Sensitivity	0.85	0.00	1.00	0.7
Specificity	0.77	1.00	0.94	0.93
Positive predictive value	0.76	–	0.71	0.74
Negative predictive value	0.86	0.88	1.00	0.91
Balanced accuracy	0.81	0.50	0.95	0.81

#### Adenocarcinoma

Pancreatic adenocarcinomas were hypoechoic (17/40) or had a mixed (23/40) echogenicity on B-mode examination. Acoustic enhancement was often evident (24/40), and peripancreatic reactivity was evident in 22 of the 40 cases. Upon CEUS examination, the adenocarcinomas showed either hyperenhancement (20/40) or hypoenhancement (19/40), (isoenhencement was evident only in 1/40 cases). Nodules diagnosed as adenocarcinomas showed mainly a homogeneous (20/40) and diffused distribution of the contrast medium (27/40). Intralesional microcirculation was evident in 28 of the 40 cases. Hypoperfused areas were evident in 21 cases. Lastly, 26 cases had no wash-out whereas the remaining 14 cases had a hypoenhancing wash-out. Interestingly, intralesional microcirculation was seen in 4 of the 5 of the adenocarcinomas showing a pseudocystic appearance.

#### Lymphoma

Pancreatic lymphomas were mainly hypoechoic (7/11) in appearance during B-mode examination while both acoustic enhancement (9/11) and peripancreatic reactivity (7/11) were often evident. On CEUS examination, lymphomas had either a hyperenhancing (7/11) or hypoenhancing (3/11) appearance, with diffused (9/11) distribution of the contrast medium. Presence of intralesional microcirculation and hypoperfused areas were evident in 6 of the 11 cases. These lesions showed a hypoenhancing wash-out phase in 9 cases, whereas lymphoma had no wash-out in 8 cases.

#### Nodular hyperplasia

Nodular hyperplasia lesions were mainly hypoechoic (15/17) on B-mode examination. Peripancreatic reactivity was evident in 8 cases. Nodular hyperplasia showed peculiar CEUS features with an isoenhancing pattern, and a diffuse and homogeneous distribution of the contrast medium. None of the cases showed a wash-out. In fact, after contrast medium administration, the lesions had the same CEUS features as the remainder of the pancreatic parenchyma cases.

#### Cysts

On B-mode examination, all the cyst were completely anechoic, and acoustic enhancement was always evident. The dimensions ranged from 0.5 to 1.1 cm in diameter. Peripancreatic reactivity was evident in 3 of the 4 cases. All the cysts were non-enhancing at CEUS examination.

#### Other benign lesions

The class “other benign lesions” was a heterogeneous class of lesions comprising seven different final cytopathological diagnoses. Consequently, OBLs did not show any characteristic B-Mode or CEUS features. Most were hyperenhancing (12/20) even if all qualitative and quantitative enhancement types were present within the group. The majority of the lesions showed a homogeneous enhancement while hypoperfused areas were almost always evident.

#### Other malignant lesions

“Other malignant lesions” was also a heterogeneous class of lesions comprising five different cytopathological diagnoses. Most of the OMLs in this category had a mixed echogenicity on B-mode examination, whereas 2 were hyperechoic. Acoustic enhancement was evident in 4 of the 6 lesions, and peripancreatic reactivity was evident in 3 of the 6. On CEUS examination, most of the OMLs (5/6) were hyperenhancing, with a diffuse distribution of the contrast medium. Intralesional microcirculation was evident in 4 of the 6 lesions, while contrast enhancing was inhomogeneous in 4 of the 6. Hypoperfused areas were evident in most (5/6) cases. Four of the 6 cases had no wash-out whereas one of the remaining cases showed a homogeneous diffused wash-out and the other showed an inhomogeneous diffused wash-out.

## Discussion

Feline pancreatic diseases are still a diagnostic challenge and, therefore, the scope to also include CEUS in the diagnostic algorithm could stand as an easy-to-follow and easy-to-use diagnostic tool for the clinician. Such a possibility has been limited until now by the almost total absence of references on this topic. Indeed, to the best of the authors' knowledge, this is the first comprehensive study describing the CEUS features of feline pancreatic diseases based on their cytopathological diagnosis. The large number of cases included in this study, especially for some cytopathological categories, makes the reported results a useful guide for the clinician in the interpretation of this ancillary examination technique.

The feline medical literature existing today mainly focuses on the description and characterization of the ultrasonographic appearance of diffused pancreatic lesions (14), whereas only a few research papers focusing on focal pancreatic lesions (15, 16) are available. In particular, this is the first study reporting the B-mode features of a large number of FPLs. Given that the CEUS features of FPLs in cats are scarcely reported in the literature, a straightforward comparison with the results of this study is not possible. On the other hand, the B-mode features of FPLs in cats have been described, and therefore the comparison between the results of this study and the available literature will focus mainly on B-mode.

Pancreatic adenocarcinomas are described as mainly hypoechoic nodules, and the presence of pancreatic solitary masses exceeding 1.5 cm in diameter is considered as suggestive of adenocarcinoma (17). Adenocarcinomas showed both mixed echogenicity (23/40–57%) and hypoechogenicity (16/40–40%) in the present study. A single case of feline pancreatic adenocarcinoma was included in a previous study describing the CEUS features of pancreatic lesions in cats (8); nonetheless, the CEUS features of that particular case were not reported and no direct comparison with the results presented here are possible. The results of the present study suggest that adenocarcinomas were large, hyperenhancing or hypoenhancing lesions with evident intralesional microcirculation on CEUS examinations. Interestingly, the mean dimensions of adenocarcinomas were only significantly different to those of nodular hyperplasia. Unsurprisingly, lesion dimensions were not used for classification in any of the branches of the decision tree.

To the best of our knowledge this is the first paper reporting the CEUS features of feline pancreatic lymphoma. The B-mode features of a single case of feline pancreatic lymphoma, described as a nodular hypoechoic lesion, is reported in the literature (15). In this study hypoechogenicity was the most common B-mode feature for lymphomas but a substantial number of cases (4/11) showed a mixed echogenicity. The CEUS features of lymphomas were also highly variable and, indeed, lymphomas did not show any distinctive feature that could guide the clinician in the differentiation from other lesions. Unsurprisingly, the decision tree analysis was not able to differentiate lymphoma from the remainder cytopathological categories.

Nodular hyperplasia was the only category exhibiting peculiar features, for both B-mode and CEUS. Indeed, nodular hyperplasia lesions were mainly small hypoechoic (15/17–83%) nodules in B-mode. Upon CEUS examination, all the lesions were isoechoic to the remainder of the pancreatic parenchyma and had no wash-out. In summarizing these findings, it may be stated that these lesions “disappeared” on CEUS examination but a handful of other lesions were classed as isoenhancing. Nevertheless, all 5 OBLs showing isoenhancing had a pseudocystic appearance—a feature that was never evident in nodular hyperplasia. Only one lesion with a final diagnosis of lymphoma had both CEUS and B-mode features that resembled those of nodular hyperplasia.

An overlap between the ultrasonographic features of true pseudocysts and cystic neoplasia is reported in the literature (18). These results are confirmed by the study data and, indeed, even if most of the lesions categorized as OBL had a pseudocystic aspect, 5 adenocarcinomas also showed such a feature. Intralesional microcirculation was evident only in 3 of the 19 (15%) OBLs whereas most (80%) of the adenocarcinomas showing a pseudocystic appearance had evident intralesional microcirculation. It is the authors' opinion that such a feature could be used in a clinical setting for the differentiation between true pseudocysts and adenocarcinomas with a pseudocystic aspect. Obviously, the clinician must take in account that not only adenocarcinomas but also some benign lesions might that show such a feature.

The decision tree is a machine learning-based tool that has seldom been proposed in the veterinary medical literature for use by the clinician as a guide in interpreting both CEUS (19) and CT examinations (20, 21). In the study by Burti et al. (19), a decision tree was developed on 150 hepatic masses (used as a training set) and tested on another 35 cases (used as a test set). The reported accuracy is calculated on an external pool of cases in this approach and, therefore, might better represent the infield performances. Due to the limited number of cases (especially for some cytopathological categories), a cross-validation scheme involving the retesting of the decision tree on the same cases which it was developed was used. It should be stated at this point that the classification accuracy for new cases could be lower than the accuracy resulting from cross-validation. The same cross-validation scheme has also been used in other studies (20, 21). It is the authors' opinion that, despite the above-mentioned limitations, the proposed decision tree could act as a guide in classifying lesions based on their CEUS features.

A limitation of this study is that, for statistical purposes, some lesions (such as OMLs and cysts) were not included in the statistical analysis and some CEUS features of individual cytopathological lesions belonging to the OBL category were not described. The B-mode and CEUS features of cysts (anechoic, thin-walled structures with clear acoustic enhancement and no enhancement) are so peculiar that, even if they were not included in the statistical analysis, the description provided here might be sufficient grounds for making a final diagnosis of pancreatic cyst simply based on the B-mode and CEUS features. On the other hand, the OML category was so small and heterogeneous that no distinctive features of the lesions in this category was distinguishable. Another limitation of this study is that histopathology was not performed in any of the included patients. To date, no studies comparing the accuracy of histology and cytopathology of feline FPLs are available in the veterinary literature. On the other hand, Xenoulis (1) reported that, in case of a pancreatitis, also a negative result of histological examination should be interpreted with caution, and none of the diagnostic techniques can be totally reliable for the diagnosis of pancreatitis in cats and none of them is now considered as a gold standard method. On the other hand, more recent studies have used histopathology as gold standard method for the diagnosis of pancreatic disease in cats (3).

In such a scenario, it appears evident that the pathological characterization of focal pancreatic lesions remains a fundamental step in the diagnostic workflow of feline pancreatic pathology. Despite these limitations, the authors are deeply confident that the information reported here will be useful to the clinician in evaluating pancreatic disease in cats.

## Conclusions

The B-mode and CEUS features of 98 FPLs in cats are described in this article. Furthermore, an easy-to-follow decision tree to classify feline FPLs based on their B-mode US and CEUS examinations was developed. Nodular hyperplasia were small, hypoechoic nodules showing isoenhancement and no wash-out upon CEUS examination. Both adenocarcinomas and lymphomas were larger nodules (compared to nodular hyperplasia), with a mixed echogenicity and hyperenhancement or hypoenhancement at CEUS. Most of the benign lesions other than nodular hyperplasia (true pseudocysts), had a pseudocystic appearance and no intralesional vascularization. Lesions with a pseudocystic appearance and intralesional vascularization were most likely adenocarcinomas. Cyst were anechoic, thin-walled structures with clear acoustic enhancement and no CEUS enhancement.

## Data availability statement

The original contributions presented in the study are included in the article/supplementary material, further inquiries can be directed to the corresponding author/s.

## Ethics statement

This study was conducted respecting Italian Legislative Decree N° 26/2014 (transposing EU directive 2010/63/EU). Since the data used in this study were part of routine clinical activity, no ethical committee approval was required. Informed consent for personal data processing was obtained from the owners.

## Author contributions

SB, AZ, TB, and FB conceived the project, drafted, and revised the manuscript. GR, RO, and PB performed the CEUS examinations, and drafted and revised the manuscript. BC conducted the data analysis and revised the manuscript. All authors contributed to the article and approved the submitted version.

## Funding

The present paper was part of a project funded by a research grant from the Department of Animal Medicine, Production and Health – MAPS, University of Padua, Italy: SID- Banzato 2019 (€ 16,850; Application of deep-learning algorithms in pet animal diagnostic imaging).

## Conflict of interest

Author GR is the owner of ULTRAVET. The remaining authors declare that the research was conducted in the absence of any commercial or financial relationships that could be construed as a potential conflict of interest.

## Publisher's note

All claims expressed in this article are solely those of the authors and do not necessarily represent those of their affiliated organizations, or those of the publisher, the editors and the reviewers. Any product that may be evaluated in this article, or claim that may be made by its manufacturer, is not guaranteed or endorsed by the publisher.

## References

[1] Xenoulis PG. Diagnosis of pancreatitis in dogs and cats. J Small Anim Pract. (2015) 56:13–26. 10.1111/jsap.12274 25586803

[2] Seaman RL. Exocrine pancreatic neoplasia in the cat: a case series. J Am Anim Hosp Assoc. (2004) 40:238–45. 10.5326/0400238 15131106

[3] Törner K Staudacher M Tress U Weber CN Stadler C Grassinger JM. Histopathology and feline pancreatic lipase immunoreactivity in inflammatory, hyperplastic and neoplastic pancreatic diseases in cats. J Compar Pathol. (2020) 174:63–72. 10.1016/j.jcpa.2019.10.195 31955805

[4] Oppliger S Hartnack S Reusch CE Kook PH. Agreement of serum feline pancreas–specific lipase and colorimetric lipase assays with pancreatic ultrasonographic findings in cats with suspicion of pancreatitis: 161 cases (2008–2012). J Am Vet Med Assoc. (2014) 244:1060–5. 10.2460/javma.244.9.1060 24739116

[5] Banzato T Burti S Rubini G Orlandi R Bargellini P Bonsembiante F Zotti A. Contrast-enhanced ultrasonography features of hepatobiliary neoplasms in cats. Vet. Rec. (2020) 186:320. 10.1136/vr.105453 31582574 PMC7079193

[6] Leinonen MR Raekallio MR Vainio OM Ruohoniemi MO Biller DS O'Brien RT. Quantitative contrast-enhanced ultrasonographic analysis of perfusion in the kidneys, liver, pancreas, small intestine, and mesenteric lymph nodes in healthy cats. Am. J. Vet. Res. (2010) 71:1305–11. 10.2460/ajvr.71.11.1305 21034321

[7] Diana A Linta N Cipone M Fedone V Steiner JM Fracassi F. Contrast-enhanced ultrasonography of the pancreas in healthy cats. BMC Vet Res. (2015) 11:64. 10.1186/s12917-015-0380-2 25879918 PMC4367928

[8] Simeoni F Terragni R Rubini G Tamburro R Del Signore F Falerno I. B-Mode and contrast enhanced ultrasonography features of gastric inflammatory and neoplastic diseases in cats. Animals. 2020 10:1444. 10.3390/ani10081444 32824834 PMC7460435

[9] Rademacher N Ohlerth S Scharf G Laluhova D Sieber-Ruckstuhl N Alt M. Contrast-enhanced power and color Doppler ultrasonography of the pancreas in healthy and diseased cats. J Vet Intern Med. (2008) 22:1310–6. 10.1111/j.1939-1676.2008.0187.x 18798791

[10] Cervone M Harel M Ségard-Weisse E Krafft E. Use of contrast-enhanced ultrasonography for the detection of a feline insulinoma. JFMS Open Rep. (2019) 5:2055116919876140. 10.1177/2055116919876140 31579524 PMC6757499

[11] Ferrandis I Jakovljevic S Aprea F Corletto F. Effect of two sedative protocols and hepatosplenic disease on Doppler indices of splenic arteries in dogs: a preliminary study. Vet J. (2013) 197:712–6. 10.1016/j.tvjl.2013.03.030 23623458

[12] Jergens AE Hostetter SJ ACN. Oral cavity, gastrointestinal tract, and associated structures. In:Raskin RE, editor. Canine and Feline Cytology: a Color Atlas and Interpretation Guide. St. Louis: Elsevier (2016). p. 220–46 10.1016/B978-1-4557-4083-3.00007-3

[13] Choi US AT. Endocrine/neuroendocrine system. In:Raskin RE MD, editor. Canine and Feline Cytology: A Color Atlas and Interpretation Guide. Elsevier (2016). p. 430–52 10.1016/B978-1-4557-4083-3.00016-4

[14] Nivy R Kaplanov A Kuzi S Mazaki-Tovi M Yas E Segev G. A retrospective study of 157 hospitalized cats with pancreatitis in a tertiary care center: clinical, imaging and laboratory findings, potential prognostic markers and outcome. J Vet Intern Med. (2018) 32:1874–85. 10.1111/jvim.15317 30315665 PMC6271303

[15] Hecht S Penninck DG Keating JH. Imaging findings in pancreatic neoplasia and nodular hyperplasia in 19 cats. Vet Radiol Ultrasound. (2007) 48:45–50. 10.1111/j.1740-8261.2007.00203.x 17236360

[16] Bennett PF Hahn KA Toal RL Legendre AM. Ultrasonographic and cytopathological diagnosis of exocrine pancreatic carcinoma in the dog and cat. J Am Anim Hosp Assoc. (2001) 37:466–73. 10.5326/15473317-37-5-466 11563446

[17] Linderman MJ Brodsky EM de Lorimier LP Clifford CA Post GS. Feline exocrine pancreatic carcinoma: a retrospective study of 34 cases. Vet Compar Oncol. (2013) 11:208–18. 10.1111/j.1476-5829.2012.00320.x 22612638

[18] VanEnkevort BA O'Brien RT Young KM. Pancreatic pseudocysts in 4 dogs and 2 cats: ultrasonographic and clinicopathologic findings. J Vet Intern Med Am Coll Vet Intern Med. (1999) 13:309–13. 10.1111/j.1939-1676.1999.tb02186.x 10449220

[19] Burti S Zotti A Rubini G Orlandi R Bargellini P Bonsembiante F. Contrast-enhanced ultrasound features of malignant focal liver masses in dogs. Sci Rep. (2020) 10:1–12. 10.1038/s41598-020-63220-3 32269300 PMC7142119

[20] Burti S Zotti A Bonsembiante F Contiero B Banzato T. diagnostic accuracy of delayed phase post contrast computed tomographic images in the diagnosis of focal liver lesions in dogs: 69 cases. Front Vet Sci. (2021) 8:611556. 10.3389/fvets.2021.611556 33748206 PMC7969650

[21] Burti S Zotti A Bonsembiante F Contiero B Banzato T. A machine learning-based approach for classification of focal splenic lesions based on their CT Features. Front Vet Sci. (2022) 9:872618. 10.3389/fvets.2022.872618 35585859 PMC9108536

